# DIANA-miRPath v4.0: expanding target-based miRNA functional analysis in cell-type and tissue contexts

**DOI:** 10.1093/nar/gkad431

**Published:** 2023-06-01

**Authors:** Spyros Tastsoglou, Giorgos Skoufos, Marios Miliotis, Dimitra Karagkouni, Ioannis Koutsoukos, Anna Karavangeli, Filippos S Kardaras, Artemis G Hatzigeorgiou

**Affiliations:** DIANA-Lab, Department of Computer Science and Biomedical Informatics, Univ. of Thessaly, Lamia 35131, Greece; Hellenic Pasteur Institute, Athens 11521, Greece; DIANA-Lab, Department of Computer Science and Biomedical Informatics, Univ. of Thessaly, Lamia 35131, Greece; Hellenic Pasteur Institute, Athens 11521, Greece; DIANA-Lab, Department of Computer Science and Biomedical Informatics, Univ. of Thessaly, Lamia 35131, Greece; Hellenic Pasteur Institute, Athens 11521, Greece; Department of Pathology, Beth Israel Deaconess Medical Center, Boston, MA, USA; Harvard Medical School, Boston, MA, USA; Broad Institute of MIT and Harvard, Cambridge, MA, USA; DIANA-Lab, Department of Computer Science and Biomedical Informatics, Univ. of Thessaly, Lamia 35131, Greece; DIANA-Lab, Department of Computer Science and Biomedical Informatics, Univ. of Thessaly, Lamia 35131, Greece; Hellenic Pasteur Institute, Athens 11521, Greece; DIANA-Lab, Department of Computer Science and Biomedical Informatics, Univ. of Thessaly, Lamia 35131, Greece; Hellenic Pasteur Institute, Athens 11521, Greece; DIANA-Lab, Department of Computer Science and Biomedical Informatics, Univ. of Thessaly, Lamia 35131, Greece; Hellenic Pasteur Institute, Athens 11521, Greece

## Abstract

DIANA-miRPath is an online miRNA analysis platform harnessing predicted or experimentally supported miRNA interactions towards the exploration of combined miRNA effects. In its latest version (v4.0, http://www.microrna.gr/miRPathv4), DIANA-miRPath breaks new ground by introducing the capacity to tailor its target-based miRNA functional analysis engine to specific biological and/or experimental contexts. Via a redesigned modular interface with rich interaction, annotation and parameterization options, users can now perform enrichment analysis on Gene Ontology (GO) terms, KEGG and REACTOME pathways, sets from Molecular Signatures Database (MSigDB) and PFAM. Included miRNA interaction sets are derived from state-of-the-art resources of experimentally supported (DIANA-TarBase v8.0, miRTarBase and microCLIP cell-type-specific interactions) or from *in silico* miRNA–target interactions (updated DIANA-microT-CDS and TargetScan predictions). Bulk and single-cell expression datasets from The Cancer Genome Atlas (TCGA), the Genotype-Tissue Expression project (GTEx) and adult/fetal single-cell atlases are integrated and can be used to assess the expression of enriched term components across a wide range of states. A discrete module enabling enrichment analyses using CRISPR knock-out screen datasets enables the detection of selected miRNAs with potentially crucial roles within conditions under study. Notably, the option to upload custom interaction, term, expression and screen sets further expands the versatility of miRPath webserver.

## Graphical Abstract

**Graphical Abstract:**
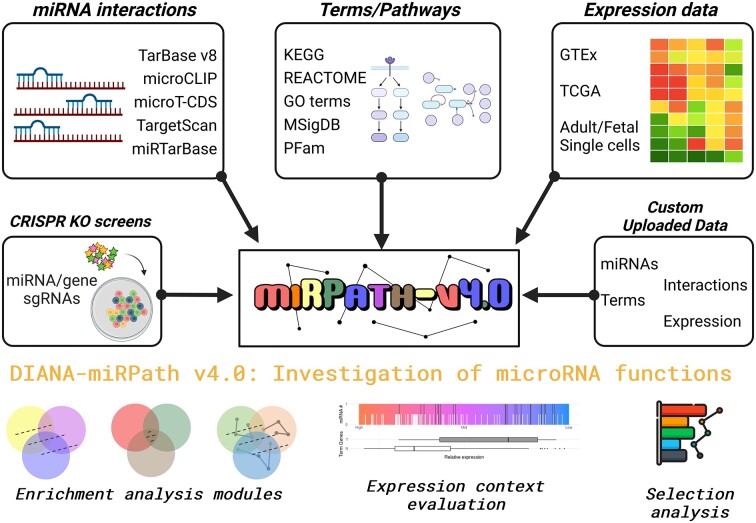

## INTRODUCTION

microRNAs (miRNAs) (1) are central post-transcriptional modulators of gene expression and protein production that exhibit abundant yet distinct expression profiles across tissues and cell-types. Robust high throughput methods directly capture functional miRNA Recognition Elements (MREs) for one or more miRNAs in the 3’ untranslated region (UTR) and/or the coding (CDS) sequence of most transcripts, providing valuable insights into miRNA regulation of most biological processes. The relative abundance of miRNAs and their targets creates unique RNA landscapes in different cell types, tissues and conditions, in which distinct miRNA regulatory roles are exerted.

*De novo* miRNA target prediction methods (2,3) and collections of miRNA interactions (4) supported by robust high-throughput experimental methods (5–7) paved the way for the development of applications conducting target-based analysis of miRNA functions (8–10). Previous versions of DIANA-miRPath (11) provided a statistical framework for evaluation of combined miRNA effects on Gene Ontology (GO) terms and KEGG pathways, based on predicted and experimentally supported targets.

**Figure 1.**
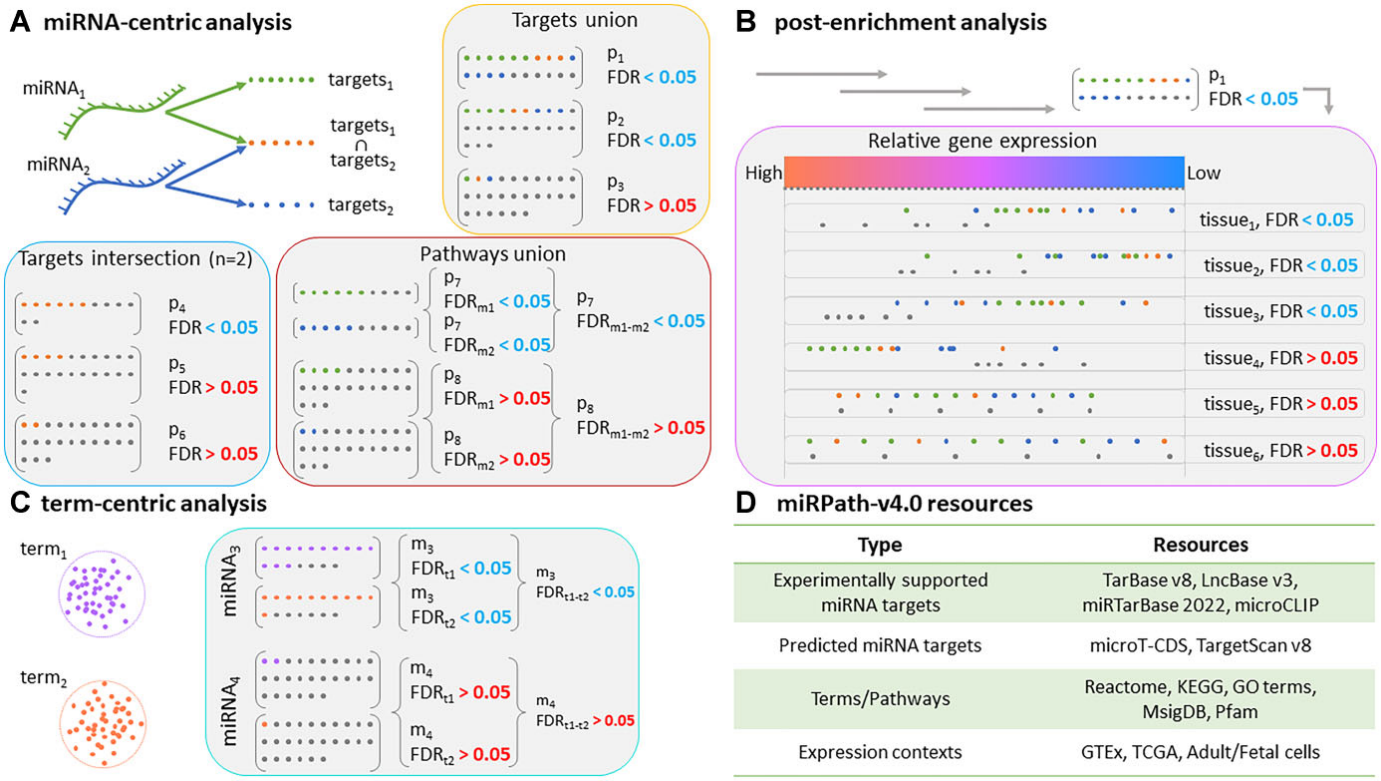
Primary DIANA-miRPath v4.0 analysis modules. (**A**) In miRNA-centric analysis, miRPath v4.0 evaluates term overrepresentation in targets of the input miRNAs, via one-sided Fisher's exact test. The Targets Union module tests the combined effect of all miRNAs. Identification of *n* miRNAs compensating each other's function by targeting the same mRNAs within specific terms (n, user-defined) can be performed under Targets Intersection module. To highlight potent term regulators, Pathways Union module assesses the individual significance of each input miRNA against each term and *a posteriori* combines separate p-values into a merged per-term significance level. (**B**) Significantly enriched terms obtained through miRNA-centric analysis can be further investigated, to uncover cell types and healthy/cancerous tissues where the expression of miRNA–targeted genes is shifted considerably relative to non-targeted genes. One-sided MWU tests are the default option, enabling the detection of instances where targets are expressed in lower levels; however the miRPath engine accommodates various exploratory scenarios, also permitting KS tests and two-sided testing. (**C**) Under term-centric analysis, miRPath v4.0 accepts as input one or multiple terms of interest and utilizes overrepresentation statistics in sets of miRNA targets, to identify miRNAs with significantly more targets in terms of interest than would be expected by chance. In the case of more than one input term, each miRNA-term combination is assessed separately and significance levels are combined per-miRNA, similarly to Pathways union module in miRNA-centric analysis (A). (**D)** Resources of miRNA interactions, terms and expression contexts that are currently integrated. miRPath v4.0 also supports analyses with user-provided data.

DIANA-miRPath v4.0 is the first relevant application to integrate robust experimental (12,13) and predicted (2,3) interactions with phenotypic and gene expression contexts, towards tailored miRNA functional analyses. In the current version, analyses have been dissected into separate modules that each answer different biological questions (Figure 1). Upon providing one or multiple miRNAs and selecting resources of miRNA interactions and terms/pathways/ontologies (hereafter, ‘terms’), enrichment analysis can be performed using the miRNA targets union, intersections, or more complex designs (Figure 1A). miRPath v4.0 extends the functionality of previous versions towards context-specific interrogations by applying nonparametric analysis of targeted and non-targeted term components in cell types, tissues and diseases. Nonparametric testing of log-scaled gene expression fold changes has been successfully utilized in the past to assess the functional efficacy of miRNA binding events (2,7). In the current scenario, it is repurposed to enable the identification of contexts and states in which the distribution of the targets is found repressed/de-regulated, transforming miRPath analyses from context-agnostic to context-aware endeavours (Figure 1B). Distribution testing is performed against internal libraries of expression sets, including tissues (healthy or cancerous) and cell types (adult or fetal), outputting results per term, sorted by level of significance. The option to also upload and use custom datasets expands the applicability of context-specific testing even more. In the absence of specific miRNAs of interest, explorations focused instead on terms can be initiated via Term-centric analysis, uncovering potential common miRNA regulators across one or multiple terms (Figure 1C). A plethora of interaction, term and context options are available by default in DIANA-miRPath v4.0 (Figure 1D).

Potential biases in miRNA functional analysis, relevant to the multiplicity of miRNA targeting, as well as the 3'-UTR length and MRE differences among transcripts within terms, have been discussed in the past (11,14). In DIANA-miRPath v3.0, permutation-based testing, which is computationally intensive and yields non-granular p-values, was provided as an alternative testing method in order to close this gap. In the current version, it is replaced by Wallenius non-central hypergeometric testing (15), parameterized to account for bias in targeting events among tested terms.

DIANA-miRPath v4.0 provides a novel module that caters miRNA-specific functional analysis of positive/negative selection results obtained using CRISPR knock-out screen libraries in stressors of interest. Via the relevant module, users can provide tab-separated results from MAGeCK analysis (RRA or MLE) of libraries targeting both genes and miRNAs, such as GeCKO v2.0 human (16,17), and obtain significantly enriched terms, miRNAs and targets that are positively/negatively selected in conditions under study.

MIENTURNET (18), miRPathDB 2.0 (19) and miR2GO (20) are three webservers that are also devoted to miRNA functional analysis. In MIENTURNET users can also produce miRNA-mRNA interaction networks however a term-based analysis is not supported. miRPathDB 2.0 provides a set of downstream information for the queried miRNA (e.g. stem-loop, mature and seed sequence) for both miRBase and miRCarta annotations but supports only human and mouse species and single miRNA queries. On the contrary, the miRNA-centric module of miRPath-v4.0 performs functional enrichment analysis allowing as input both single and multiple miRNAs, to uncover the potential combinatorial effects of miRNAs in terms. miR2GO provides an interface for miRNA functional analysis that also evaluates the functional effects of mutations in miRNA seeds. Even though this feature is not catered as a built-in miRPath capacity, users may still conduct such investigations by making use of the Upload Dashboard module.

miRPath-v4.0 extends the functionality of its previous version (DIANA-miRPath-v3.0) and offers numerous improvements over all the aforementioned implementations. miRPath-v4.0 provides the largest set of resources for both experimentally supported and predicted miRNA-gene interactions, biological terms and pathways and gene expression profiles. Importantly, it is the only method to our knowledge to incorporate gene expression profiles with single-cell resolution, as demonstrated in Supplementary Note S1, Supplementary Figures S1–S4, and Supplementary Tables S1–S3. Two newly introduced key advantages of miRPath-v4.0 are the dedicated workflow for miRNA-centric functional analysis of CRISPR-KO Screen data, for which a specific use-case scenario (21) is provided in Supplementary Note S2 and Supplementary Tables S4–S5, and the Upload Dashboard, where users can unlock the full capacity of miRPath with custom annotations (i.e. miRNAs, genes, terms) for the species/phenotypes of their choice. The case that specific terms are of interest, yet no miRNAs are provided to initiate a miRNA-centric analysis is shown in Supplementary Note S3 and Supplementary Tables S6–S7, which provide a Term-centric miRPath application. The latest version of miRPath is the only relevant implementation to provide support for both miRBase and MirGeneDB miRNA annotation schemes. It hosts the largest number of supported species, offers an option to account for bias in targeting events and provides a completely redesigned, intuitive interface to enhance user experience and enable its ingenious use.

## METHODS AND RESULTS

### Statistical methods

#### Main analysis engine

Enrichment analysis is performed by parameterizing function *kegga()* from *limma* package (22) to user-selected resources. Depending on the scenario, the union or intersection(s) of miRNA targets is calculated and subjected to testing. In the case of Pathways union, the targets of each miRNA are tested separately and significantly enriched instances are merged per-term using Fisher's method (23), as implemented in *metap* package. The option to account for targeting differences among tested terms through Wallenius non-central hypergeometric testing is integrated using packages *GOseq* (15) and *BiasedUrn*.

#### Context specific capacities

Post-enrichment analysis compares miRNA–targeted versus non-targeted pathway components across a selected context set (Section ‘Data collections’ below) using either a Mann Whitney U test (default) or a two-sample Kolmogorov-Smirnov test, as implemented in base R stats package (functions *wilcox.test()* and *ks.test()*), to assess difference in the mean ranks of miRNA–targeted and non-targeted genes, or if they are drawn from the same distribution, respectively. By default, one-tailed tests are used, in order to assess the repressive effect of miRNAs, however two-sided testing can also be selected to accommodate, for example, scenarios where the abundance of miRNAs under question is limited.

#### miRNA-centric analysis of phenotype selection screens

Primary term analysis of the top positively or negatively selected miRNAs (user-defined) is performed taking the union of their targets and employing hypergeometric testing, as described in, ‘Main analysis engine’. For significantly enriched pathways, two downstream options are provided: the default option performs competitive, rank-based CAMERA testing (proposed and described by Wu and Smyth (24)) to identify significant left/right trends of targets within the opposite ranking (e.g. for positively selected miRNAs the negative ranking of genes is assessed); the second option also requires setting the number of top opposite-ranked genes and performs one-sided Fisher's exact test to evaluate the enrichment on the rank's extreme in miRNA targets.

### Data collections

Gene identifiers were retrieved via Biomart from Ensembl v102 (25). miRNA annotations were retrieved from miRBase v22.1 (26) and also matched to instances from MirGeneDB v2.1 (27).

Experimentally verified interactions were retrieved from reference resources DIANA-TarBase v8 (12), DIANA-LncBase v3 (28), and miRTarBase 2022 (13). Context-aware interaction sets were obtained by analysing publicly available CLIP-Seq datasets in 35 human and 6 mouse tissues with microCLIP (7). Predicted interactions were obtained directly from TargetScan 8 and by performing *de novo* DIANA-microT-CDS executions (3) on Ensembl v102 and miRBase v22.1 annotation. microT interactions include for the first time the capacity to query host targets for virally encoded miRNAs regarding 20 viruses infecting human (EBV, KSHV, HSV1-2, HCMV, SFV, HBV, HIV1, HHV6B, MCPV, JCV, BKV, SV40 and TTV), mouse (MGHV, MCMV) and chicken (MDV1-2, HVT, ILTV). For TarBase, LncBase, miRTarBase and TargetScan, gene IDs were converted to Ensembl v102 using Ensembl ID History Converter tool or Biomart (miRTarBase) and miRNA names were manually converted to miRBase v22.1. Supplemental confidence information, including annotation on direct/indirect proof of interaction by TarBase, high/low interaction confidence annotation by miRTarBase, and interaction scores by target prediction tools, was retained for collections.

Annotation of GO terms (29,30) against Gene IDs was retrieved through Ensembl. Pathview package (31) was used to retrieve KEGG (32)-to-Entrez Gene ID relationships, which were annotated to Ensembl Gene IDs (Biomart). Reactome (33) pathway annotations were retrieved and Ensembl Gene IDs were retained and converted to Ensembl v102. Expertly curated Molecular Signatures Database (MSigDB) gene sets (34,35) regarding Hallmark (H), Oncogenic, (C7), Immunologic (C8) and Cell type (C9) signatures were retrieved using the msigdbr package (36) and annotated to Ensembl Gene IDs (Biomart). Protein family annotations were retrieved from PFam (37).

Adult and fetal single-cell RNA-Seq data was collected from the Han *et al.* human cell atlas (38). Log_2_(Fold Change) (log_2_FC) gene expression values were obtained for each cell type by estimating the percent of individual cells exhibiting at least one read within each annotated cell type and contrasting it to the median of percentages of the remaining cell types. Transcripts-Per-Million (TPM-) level gene expression data were obtained for each cancer project in TCGA (39) via FireBrowse (https://firebrowse.org). Adjacent healthy and blood-derived normal samples were filtered out. Healthy tissue expression data (TPM-level) were retrieved from the GTEx Consortium (40). TPM-level data were used to denote a per-gene per-tissue log_2_FC, similarly to the single-cell data, depicting the percentage of samples presenting at least 10 TPM in a tissue relative to the median of the percentages of the remaining tissues. This metric was adopted as a balanced estimator to depict consistent and cell-/tissue-specific non-zero expression. For all three resources (single-cell atlas, TCGA, GTEx), gene names were converted to Ensembl v102 Gene IDs using Ensembl ID History Converter tool and R.

### Implementation

**Figure 2.**
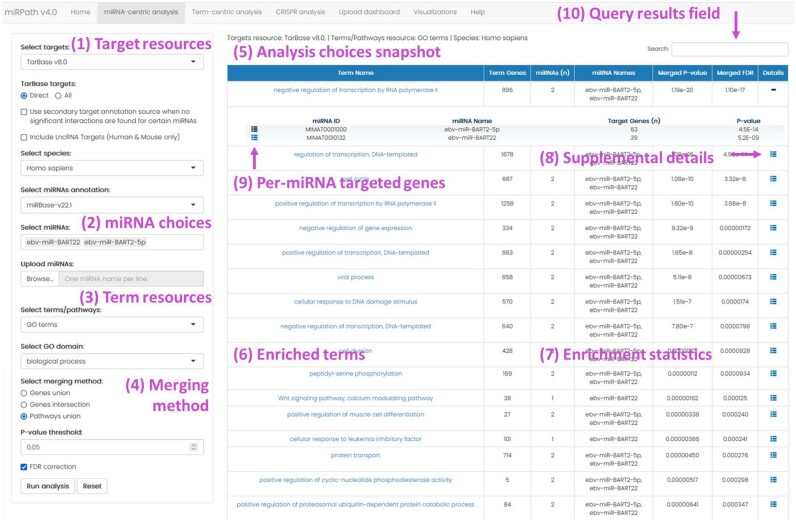
Depiction of miRNA-centric mode of DIANA-miRPath v4.0. On the left panel, users may choose among (1) different miRNA interaction resources and (2) miRNA annotations. Enrichment can be performed in sets of terms (3) after selecting the most appropriate results methodology (4) and significance thresholds. Upon conducting an analysis, (5) a brief annotation of selected options is provided. The results table is organized per-term (6), offering metrics regarding the numbers of miRNAs, total genes and targeted genes in each entry, along with the corresponding significance values (7). For terms of interest, supplementary details can be unfolded using the respective buttons (8), highlighting the interactions that yielded each specific result (9), which can also be retrieved locally. The entire results table can also be retrieved locally by Download buttons in the bottom of the page, while terms/genes/miRNAs of interest can be provided into the filter-down button (10), in order to briefly query the results table for existence of entries harbouring these terms.

The user interface of miRPath has been upgraded to provide further flexibility without sacrificing user-friendliness. Figure 2 briefly showcases the input menu and results from the tab enabling miRNA-centric functional analysis. All interaction, term and context resources included in DIANA-miRPath v4.0 were converted to Mongo collections and stored locally in a MongoDB schema (v4.4.2). DIANA-miRPath v4 was set up as an R Shiny application (https://shiny.rstudio.com/), and deployed using ShinyProxy (https://www.shinyproxy.io). The data access layer is implemented using *mongolite* package. The presentation layer relies primarily on packages *shiny*, *shinyalert*, *shinyjs*, *markdown* (41), tippy, while visualizations are created making use of packages *ggplot2* (42), *limma*, *Pathview* (31) and *pheatmap* (v1.0.12, Kolde, 2019). R Markdown was used to create a Help Section, while all functionalities requiring user input are accompanied with example data to further aid user experience.

## DISCUSSION

DIANA-miRPath v4.0 constitutes an upgrade both in terms of interface, which now holds a modular application-like character, and of content. Available visualization options were also expanded. Importantly, a User Upload Dashboard is introduced to further augment tailored use and flexibility. By it, the full arsenal of miRPath modules is unlocked for application with custom, user-provided annotations e.g. non-model organisms, modified/edited miRNAs, or even other non-coding RNAs. DIANA-miRPath v4.0 constitutes a valuable resource, delivering numerous computational methods to initiate or consolidate miRNA-related investigations.

## DATA AVAILABILITY

DIANA-miRPath is freely available, without registration or login, at http://www.microrna.gr/miRPathv4.

## Supplementary Material

gkad431_Supplemental_File
