# Supplementary material for: DIANA-miRPath v4.0: expanding target-based miRNA functional analysis in cell-type and tissue contexts
Source: Nucleic Acids Res. 2023 Jun 1;51(W1):W154–9. doi: 10.1093/nar/gkad431 (PMC10320185; doi:10.1093/nar/gkad431)
Supplement: gkad431_Supplemental_File [file gkad431_supplemental_file.pdf]

Supplementary data:

DIANA-miRPath v4.0: Expanding target-based miRNA functional analysis in cell-type and tissue contexts

Spyros Tastsoglou *et al.*

Supplementary Notes

Supplementary Note 1. Cell-type-level analysis of miR-17~92 cluster

The well-studied miR-17~92 cluster, which spans 6 polycistronically transcribed miRNAs, has been documented to exert regulatory roles in a wide range of processes, including development, differentiation, cell proliferation and the immune response, while its dysregulation has been extensively associated with cancers, developmental abnormalities, cardiovascular and neurodegenerative disease (rev. in (1)).

Using miRPath miRNA-centric module, we sought out to identify miR-17~92-regulated pathways. The dominant mature forms of the cluster (i.e., miR-17-5p, miR-18a-5p, miR-19a-3p, miR-19b-3p, miR-20a-5p and miR-92a-3p) were provided for Genes union analysis using microT-CDS targets and Reactome pathways. Subsequently, we used post-enrichment analysis to assess expression shifts of the cluster's targets, within pathways, in fetal and adult cell type sets. Briefly, post-enrichment analysis yields p-values (FDR-adjusted) of the significance of expression change for the miRNA-targeted term components, relative to non-targeted term components, per context (e.g., miRPath internal cell types, healthy tissues or cancer types, or user-provided contexts). Identifying a significant change in a specific context could indicate that the input miRNAs are exerting their regulatory role in this term and post-transcriptionally affect the targeted genes/terms within this context. Post-enrichment analysis also provides visualizations comparing the expression distribution of genes targeted by the miRNAs of interest to that of non-targeted genes. In their top panel, white vertical lines denote genes participating in the biological term of interest that are not targeted by the input miRNAs. The position of the white lines (genes) indicates their expression level/ranking in the tissue/cell/context of interest. Black lines indicate genes targeted harboring interactions with one or more of the user-selected miRNAs with the genes. The number of the different user-selected miRNAs that target a particular gene is marked by the height of the black line. For instance, when a black lines height reaches number 3 in the y axis, the respective gene is targeted by three of the user-defined miRNAs. In the bottom panel of the post-enrichment analysis visualizations, two horizontal box plots denote the expression levels of the targeted ("T", top box plot in grey) *versus* non-targeted ("N", bottom box plot in white) genes that participate in the biological term of interest. Post-enrichment analysis was performed against fetal and adult cell types, derived from single cell RNA-Seq analysis (2), utilizing one-tailed Mann Whitney U tests (0.05 FDR threshold).

The top significantly enriched term from the pathway analysis (FDR = 1E-07) was Membrane Trafficking (**Supplementary Table 1**). Membrane trafficking events have been shown to be integral components in numerous immune processes, including among others the signaling by pattern recognition receptors and Toll-like receptors, cell polarization, antigen-B-cell-receptor endocytosis, the MHC class II maturation and T-cell-receptor signaling (3-5). Among 164 fetal and 256 adult cell types in total, targeted term members were significantly shifted towards lower values in 38 and 62 cell types respectively (**Supplementary Figures 1-2**, **Supplementary Tables 2-3**), indicating potential regulatory roles of miR-17~92 cluster on membrane trafficking in these cells. Notable fractions of the significant cell types are cells of the innate and adaptive immune response (multicolored-bars in **Supplementary Figures 1-2**). The miRNA cluster's high expression in embryonic cells and role in developmental processes is well-established (6-8), while, according to the DIANA miRNA Tissue Expression Database, miR-17~92 expression appears more than 2-fold higher in fetal samples compared to the adult liver samples (9). In **Supplementary Figures 3-4**, the expression distribution of Membrane trafficking genes targeted by miRs 17~92 is compared to that of non-targeted genes in fetal and adult B cells. In both cases, targeted genes exhibit expression levels that are clearly shifted towards the right end of the line (i.e., lower expression), while expression difference in fetal B cells appears more prominent than in adult B cells.

Supplementary Note 2. Using miRPath to identify miRNAs positively/negatively modulating Cancer Stem Cells in an osteosarcoma genome-wide CRISPR knockout screen

In order to identify Cancer Stem Cell (CSC) modulators in osteosarcoma, Wang Y. *et al*. have realized a pooled CRISPR knockout screen dataset (10). Briefly, after identifying pluripotency transcription factor Oct4 as a critical regulator of osteosarcoma CSC-like properties, they induced a system of Enhanced Green Fluorescent Protein (EGFP) driven by Oct4 enhancers (eOct4-EGFP) in MG63 osteosarcoma cells (which are cells with low basal levels of Oct4) and used them for a genome-wide CRISPR screen (GeCKO v2 library). After culture, sequencing of single-guide RNA (sgRNA) populations in the resulting eOct4-$EGFP^{high}$ and eOct4-$EGFP^{-}$ cells was performed. Downstream analysis focused on the mechanistic study of transcription factor KLF11, which was the top target of sgRNAs enriched in $EGFP^{high}$ cells (i.e., a negative regulator of CSCs), as identified employing MAGeCK approach (11).

We used MAGeCK results from this study on miRPath CRISPR analysis module, to conduct a pathway analysis analysis of the screen with a focus on positively/negatively selected miRNAs. Reactome pathways and microT-CDS interactions (0.7 score threshold) were chosen as input resources to explore miRNAs with negative/positive regulatory functions for CSCs. In order to identify negative miRNA regulatory activity for CSCs, we selected the top 10 positively selected miRNAs (i.e., enriched in $EGFP^{high}$ cells, $rank^{positive}$ range: 5-214, incl. non-miRNA genes), as well as the top 100 negatively selected genes (maximum $rank^{negative}$ = 110, incl. miRNA genes) for miRNA target overrepresentation analysis in positive CSC regulator mRNAs. Conversely, to detect miRNA functions with positive roles for CSC propagation, the top 10 negatively selected miRNAs (i.e., depleted in $EGFP^{high}$ cells, $rank^{negative}$ range: 10-100, incl. non-miRNA genes) and the top 100 positively selected genes (maximum $rank^{positive}$ = 103, incl. miRNA genes) were chosen. miRPath analysis pinpointed a number of positively/negatively selected miRNAs as potential (co-)modulators in the establishment/abolishment of CSC-like properties in osteosarcoma.

Using the negative miRNA regulators of CSCs, 5 significantly enriched terms were identified in which the 100 top positively selected CSC regulators were also overrepresented at FDR < 0.05 (**Supplementary Table 4**). In this example, miRs 214-3p, 214-5p, 4447, 4776-3p, 4776-5p, 6069, 6812-5p, 6891-3p and 6891-5p, which appear as negative regulators of CSC, significantly enrich Signaling by NTRK1 pathway, and have predicted interactions with genes PIK3CA, RHOA, STAT3 and TRIB1 which appear as positive CSC regulators. The tumorigenic effects of dysregulated NTRK receptor family signaling, extending to ligand-independent pathway activation (12), have been documented in a number of cancers (13). Particularly, pro-apoptotic and anti-proliferative effects of TRKA signaling inhibition have been reported in human, mouse and canine osteosarcoma studies (14). PI3K/AKT signaling is crucial for proliferation, cell survival and cell cycle regulation and could be involved in maintenance and renewal of Cancer Stem Cell osteosarcoma deposits (15).

The positive miRNA CSC regulators significantly enriched Hippo signaling (**Supplementary Table 5**). Among genes found as negative regulators of CSC (which are contained in predicted miRNA interactions of the set of positive CSC regulator miRs 1246, 18a-5p, 4472, 495-3p, 495-5p, 548x-3p, 607, 6883-3p and 922), LATS1 and LATS2 stand out as two known tumor suppressors whose inhibition has already been linked to Cancer Stem Cell state (16).

In conclusion, CRISPR analysis module builds on the results of CRISPR knockout analysis to link miRNAs and genes participating in specific pathways and terms with the condition under study.

Supplementary Note 3. Utilization of miRPath-v4.0 to identify miRNA regulators of the ECM-receptor interaction pathway

The ECM-receptor interaction pathway plays a crucial role in the regulation of various cellular processes, including cell adhesion, migration, proliferation, and differentiation. This intricate network of interactions involves the extracellular matrix (ECM), a complex structure of proteins and carbohydrates, and specific cell surface receptors. Since miRNAs and ECM both have vital roles in organogenesis during development, we sought to explore the potential interplay between them. To this end, we utilized the Term-centric module of miRPath, whose role is to find sets of miRNAs with the potential to regulate one or more biological terms.

This module accepts as input one or multiple terms of interest, resources for miRNA-gene interactions (e.g., TarBase, TargetScan, microT-CDS), a resource for biological terms (e.g., KEGG, Reactome), a miRNA annotation scheme (i.e., miRBase, MiRGeneDB) and a p-value threshold (with the option for FDR correction) to control the significance level of the results. The term "ECM-receptor interaction" from KEGG was provided to miRPath for Term-centric analysis using TarBase direct targets and the miRBase 22.1 annotation at a 0.05 FDR-adjusted significance threshold.

The analysis delivered four significant miRNAs with the potential to regulate ECM-receptor interaction (**Supplementary Table 6**). Interestingly, a recently published study by Viktoria Wagner *et al.* (Nature Biotechnology, 2023) (17) manifested miR-29 as a major regulator of ECM and other ECM-associated biological processes (e.g., collagen fibril organization). The two top microRNAs enriched by the Term-centric analysis we conducted with miRPath are hsa-miR-29b-3p and hsa-miR-29c-3p. According to TarBase direct miRNA-gene interactions, both miR-29b-3p and miR-29c-3p negatively regulate the expression of 17 protein-coding genes participating in ECM-receptor signaling (**Supplementary Table 7**), many of which directly influence the formation of the extracellular matrix (i.e., COL1A1, COL4A2, COL1A2 etc.).

In conclusion, we employed the Term-centric analysis module of miRPath-v4.0 in a miRNA-agnostic manner to investigate whether the KEGG pathway "ECM-receptor interaction" could possibly be under regulation by miRNAs using the set of direct experimentally supported miRNA-gene interactions provided by TarBase.

Supplementary Tables

Supplementary Table 1. Top Reactome pathways enriched by miR-17~92 polycistronic cluster.

Pathway	Targeted over total genes	miRNA Names	P-value	FDR
Membrane Trafficking	205/653	17-5p, 18a-5p, 19a-3p, 19b-3p, 20a-5p, 92a-3p	6E-11	1E-07
Neuronal System	145/438	17-5p, 18a-5p, 19a-3p, 19b-3p, 20a-5p, 92a-3p	9E-10	1E-06
Generic Transcription Pathway	363/1372	17-5p, 18a-5p, 19a-3p, 19b-3p, 20a-5p, 92a-3p	9E-08	6E-05
Clathrin-mediated endocytosis	59/148	17-5p, 18a-5p, 19a-3p, 19b-3p, 20a-5p, 92a-3p	1E-07	6E-05
RNA Polymerase II Transcription	390/1509	17-5p, 18a-5p, 19a-3p, 19b-3p, 20a-5p, 92a-3p	4E-07	2E-04
Gene expression (Transcription)	424/1661	17-5p, 18a-5p, 19a-3p, 19b-3p, 20a-5p, 92a-3p	5E-07	2E-04
Post-translational protein modification	397/1552	17-5p, 18a-5p, 19a-3p, 19b-3p, 20a-5p, 92a-3p	1E-06	4E-04
Signaling by TGFB family members	43/104	17-5p, 18a-5p, 19a-3p, 19b-3p, 20a-5p, 92a-3p	2E-06	5E-04
Downregulation of SMAD2/3:SMAD4 transcriptional activity	15/23	17-5p, 18a-5p, 19a-3p, 19b-3p, 20a-5p, 92a-3p	5E-06	1E-03
Synthesis of PIPs at the plasma membrane	27/57	17-5p, 18a-5p, 19a-3p, 19b-3p, 20a-5p, 92a-3p	7E-06	2E-03
Circadian Clock	31/70	17-5p, 18a-5p, 19a-3p, 19b-3p, 20a-5p, 92a-3p	9E-06	2E-03
Intracellular signaling by second messengers	107/350	17-5p, 18a-5p, 19a-3p, 19b-3p, 20a-5p, 92a-3p	1E-05	2E-03
Vesicle-mediated transport	210/780	17-5p, 18a-5p, 19a-3p, 19b-3p, 20a-5p, 92a-3p	2E-05	2E-03
Transmission across Chemical Synapses	90/287	17-5p, 18a-5p, 19a-3p, 19b-3p, 20a-5p, 92a-3p	2E-05	2E-03
Signaling by Receptor Tyrosine Kinases	150/528	17-5p, 18a-5p, 19a-3p, 19b-3p, 20a-5p, 92a-3p	2E-05	2E-03
Signaling by NTRKs	50/137	17-5p, 18a-5p, 19a-3p, 19b-3p, 20a-5p, 92a-3p	2E-05	2E-03
SUMOylation of intracellular receptors	17/30	17-5p, 18a-5p, 19a-3p, 19b-3p, 20a-5p, 92a-3p	2E-05	2E-03
Signaling by TGF-beta Receptor Complex	32/75	17-5p, 18a-5p, 19a-3p, 19b-3p, 20a-5p, 92a-3p	2E-05	2E-03
G-protein mediated events	26/57	17-5p, 18a-5p, 19a-3p, 19b-3p, 20a-5p, 92a-3p	2E-05	3E-03

Supplementary Table 2. Genes targeted by miR-17~92 cluster enrich Membrane Trafficking pathway and are significantly skewed towards lower expression – relative to the non-targeted members of Membrane Trafficking – in the following fetal cell types.

Context	FDR
Antigen presenting cell (RPS high)|Fetal liver	3E-08
CB CD34+|Fetal liver	5E-08
B cell|Fetal liver	6E-08
T cell|Fetal liver	4E-05
Erythroid progenitor cell (RP high)|Fetal liver	4E-05
CB CD34+|Fetal intestine	7E-05
T cell|Fetal female gonad	9E-05
Dendritic cell|Fetal female gonad	1E-04
T cell|Fetal intestine	1E-04
Primordial germ cell|Fetal eyes	1E-04
Erythroid cell|Fetal female gonad	2E-04
Neutrophil (RPS high)|Fetal liver	3E-04
Monocyte|Fetal liver	3E-04
Dendritic cell|Fetal liver	1E-03
T cell|Fetal thymus	2E-03
Stromal cell|Fetal pancreas	3E-03
T cell|Fetal pancreas	3E-03
Fetal epithelial progenitor|Fetal skin	3E-03
Neutrophil|Fetal liver	4E-03
Erythroid cell|Fetal liver	5E-03
Endothelial cell (APC)|Fetal pancreas	6E-03
Smooth muscle cell|Fetal female gonad	8E-03
Neutrophil (RPS high)|Fetal spinal cord	0.011
Dendritic cell|Fetal kidney	0.013
Primordial germ cell|Fetal female gonad	0.016
Fetal epithelial progenitor|Fetal brain	0.017
Fetal epithelial progenitor|Fetal muscle	0.017
Smooth muscle cell|Fetal skin	0.018
Fetal mesenchymal progenitor|Fetal rib	0.018
Proliferating T cell|Fetal lung	0.023
Erythroid cell|Fetal stomach	0.031
Monocyte|Fetal pancreas	0.034
Primordial germ cell|Fetal muscle	0.034
Dendritic cell|Fetal pancreas	0.034
Dendritic cell|Fetal thymus	0.038
T cell|Fetal adrenal gland	0.041
Erythroid cell|Fetal eyes	0.044
Fetal stromal cell|Fetal lung	0.0498

Supplementary Table 3. Genes targeted by miR-17~92 cluster enrich Membrane Trafficking pathway and are significantly skewed towards lower expression – relative to the non-targeted members of Membrane Trafficking – in the following adult cell types.

Context	FDR
B cell|Adult bone marrow	4E-07
T cell|Adult adrenal gland	4E-06
Dendritic cell|Adult bone marrow	4E-06
B cell|Adult sigmoid colon	9E-06
Mast cell|Adult transverse colon	2E-05
Fasciculata cell|Adult adrenal gland	5E-05
Dendritic cell|Adult peripheral blood	5E-05
Enterocyte|Adult sigmoid colon	2E-04
Stromal cell|Adult rectum	2E-04
Dendritic cell|Adult spleen	7E-04
Dendritic cell|Adult transverse colon	7E-04
B cell (Plasmocyte)|Adult peripheral blood	8E-04
T cell|Adult bone marrow	1E-03
Erythroid progenitor cell (RP high)|Adult spleen	1E-03
B cell|Adult transverse colon	1E-03
B cell (Plasmocyte)|Adult bone marrow	1E-03
Smooth muscle cell|Adult adrenal gland	1E-03
B cell (Plasmocyte)|Adult rectum	2E-03
T cell|Adult ascending colon	2E-03
Enterocyte progenitor|Adult epityphlon	2E-03
Basal cell|Adult prostate	2E-03
Enterocyte progenitor|Adult sigmoid colon	3E-03
T cell|Adult bladder	3E-03
T cell|Adult heart	3E-03
Myeloid cell|Adult adrenal gland	3E-03
Smooth muscle cell|Adult cerebellum	4E-03
B cell|Adult spleen	4E-03
Monocyte|Adult bladder	4E-03
Erythroid progenitor cell (RP high)|Adult bone marrow	5E-03
T cell|Adult spleen	5E-03
T cell|Adult peripheral blood	5E-03
B cell|Adult liver	6E-03
Epithelial cell|Adult sigmoid colon	6E-03
B cell|Adult rectum	6E-03
Enterocyte progenitor|Adult transverse colon	6E-03
Erythroid cell|Adult bone marrow	7E-03
Dendritic cell|Adult prostate	8E-03
Smooth muscle cell|Adult temporal lobe	9E-03
Enterocyte progenitor|Adult ascending colon	1E-02
Dendritic cell|Adult duodenum	1E-02

B cell (Plasmocyte)|Adult sigmoid colon	1E-02
T cell|Adult transverse colon	1E-02
Epithelial cell (intermediated)|Adult ureter	1E-02
Stromal cell|Adult adrenal gland	0.011
T cell|Adult sigmoid colon	0.011
Stromal cell|Adult sigmoid colon	0.011
Monocyte|Adult peripheral blood	0.011
Neutrophil (RPS high)|Adult bone marrow	0.012
Macrophage|Adult adrenal gland	0.013
Antigen presenting cell (RPS high)|Adult bone marrow	0.014
Monocyte|Adult cerebellum	0.016
B cell|Adult ascending colon	0.021
Monocyte|Adult heart	0.021
T cell|Adult epityphlon	0.021
T cell|Adult ileum	0.026
B cell (Plasmocyte)|Adult transverse colon	0.031
T cell|Adult rectum	0.038
Sinusoidal endothelial cell|Adult bladder	0.041
Dendritic cell|Adult ileum	0.041
B cell|Adult peripheral blood	0.042
Epithelial cell|Adult stomach	0.049
Enterocyte progenitor|Adult rectum	0.0497

Supplementary Table 4. miRPath CRISPR analysis of top 10 negative regulator miRNAs in osteosarcoma Cancer Stem Cells.

Pathway	Targeted over total genes	CSC-negative miRNAs	FDR	CSC-positive selected genes (n)	FDR selection
Signaling by NTRK1 (TRKA)	46/118	214-3p, 214-5p, 4447, 4776-3p, 4776-5p, 6069, 6812-5p, 6891-3p, 6891-5p	0.0092	PIK3CA, RHOA, STAT3, TRIB1 (4)	0.0061
Signaling by NTRKs	50/137	214-3p, 214-5p, 3141, 4447, 4776-3p, 4776-5p, 6069, 6812-5p, 6891-3p, 6891-5p	0.018	PIK3CA, RHOA, STAT3, TRIB1 (4)	0.0061
PI3K/AKT activation	7/9	214-3p, 4447, 4776-3p, 6812-5p, 6891-3p, 6891-5p	0.035	PIK3CA, RHOA (2)	0.015
Signaling by Receptor Tyrosine Kinases	170/528	214-3p, 214-5p, 3141, 4447, 4485-3p, 4776-3p, 4776-5p, 6069, 6812-5p, 6891-3p, 6891-5p	0.0004	PIK3CA, RHOA, STAT3, TRIB1, YAP1 (5)	0.037
Developmental Biology	335/1245	214-3p, 214-5p, 3141, 4447, 4454, 4485-5p, 4776-3p, 4776-5p, 6069, 6812-5p, 6891-3p, 6891-5p	0.033	YAP1, NANOG, NFASC, KRTAP10-1, RHOA, PIK3CA, NFASC, STAT3 (7)	0.037

Supplementary Table 5. miRPath CRISPR analysis of top 10 positive regulator miRNAs in osteosarcoma Cancer Stem Cells.

Pathway	Targeted over term genes	CSC-positive miRNAs	FDR	CSC-negative selected genes (n)	FDR Selection
Signaling by Hippo	18/22	1246, 18a-5p, 4472, 495-3p, 495-5p, 548x-3p, 607, 6883-3p, 922	0.015	LATS1, LATS2, AMOTL2 (3)	0.024

Supplementary Table 6: Four significant miRNAs with the potential to regulate ECM-receptor signaling.

Pathway	miRNA Name	miRNA Targets	Target Genes (n)	P-value	FDR
ECM-receptor interaction	hsa-miR-29b-3p	1275	17	0.0001	0.044
ECM-receptor interaction	hsa-miR-29c-3p	1256	17	0.0001	0.044
ECM-receptor interaction	hsa-miR-518c-5p	160	6	0.0001	0.044
ECM-receptor interaction	hsa-miR-539-5p	95	5	0.0001	0.044

Supplementary Table 7: Experimentally supported miRNA-gene interactions derived from TarBase. These protein-coding genes participate in the ECM-receptor interaction pathway and are compiled by direct experimental techniques.

miRNA ID	miRNA Name	Target Gene IDs	Target Gene Names
MIMAT0000100	hsa-miR-29b-3p	ENSG00000058085	LAMC2
MIMAT0000100	hsa-miR-29b-3p	ENSG00000082781	ITGB5
MIMAT0000100	hsa-miR-29b-3p	ENSG00000091409	ITGA6
MIMAT0000100	hsa-miR-29b-3p	ENSG00000108821	COL1A1
MIMAT0000100	hsa-miR-29b-3p	ENSG00000134871	COL4A2
MIMAT0000100	hsa-miR-29b-3p	ENSG00000135862	LAMC1
MIMAT0000100	hsa-miR-29b-3p	ENSG00000137801	THBS1
MIMAT0000100	hsa-miR-29b-3p	ENSG00000137809	ITGA11
MIMAT0000100	hsa-miR-29b-3p	ENSG00000142156	COL6A1
MIMAT0000100	hsa-miR-29b-3p	ENSG00000142173	COL6A2
MIMAT0000100	hsa-miR-29b-3p	ENSG00000150093	ITGB1
MIMAT0000100	hsa-miR-29b-3p	ENSG00000161638	ITGA5
MIMAT0000100	hsa-miR-29b-3p	ENSG00000163359	COL6A3
MIMAT0000100	hsa-miR-29b-3p	ENSG00000164692	COL1A2
MIMAT0000100	hsa-miR-29b-3p	ENSG00000187498	COL4A1
MIMAT0000100	hsa-miR-29b-3p	ENSG00000188153	COL4A5
MIMAT0000100	hsa-miR-29b-3p	ENSG00000196569	LAMA2

Supplementary Figures

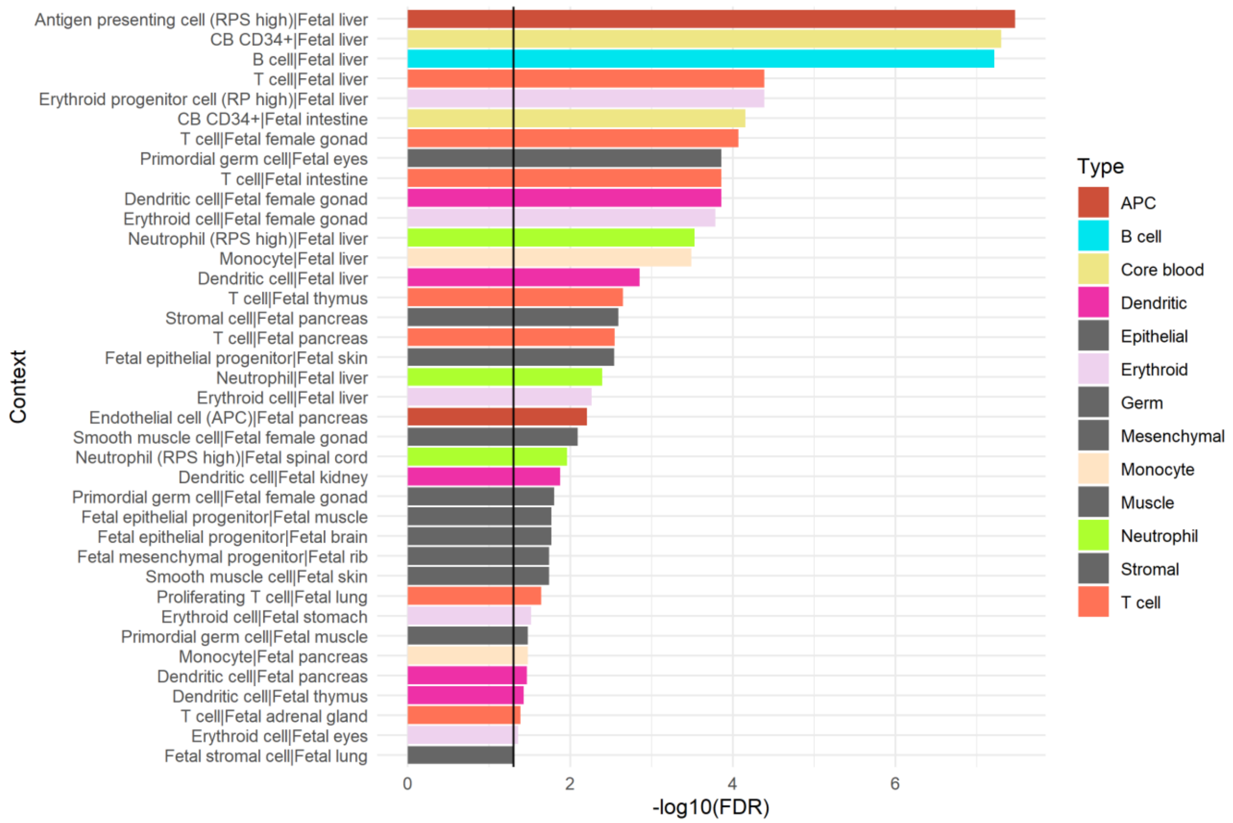

Supplementary Figure 1. Fetal cell types in which membrane trafficking targets of miR-17~92 cluster are significantly less expressed relative to non-targeted genes (Supplementary Note 1, Supplementary Tables 1-2). The vertical line signifies the 0.05 FDR threshold (one-tailed MWU test). Colored bars belong to cell types related to immune response (APC, Antigen Presenting Cell).

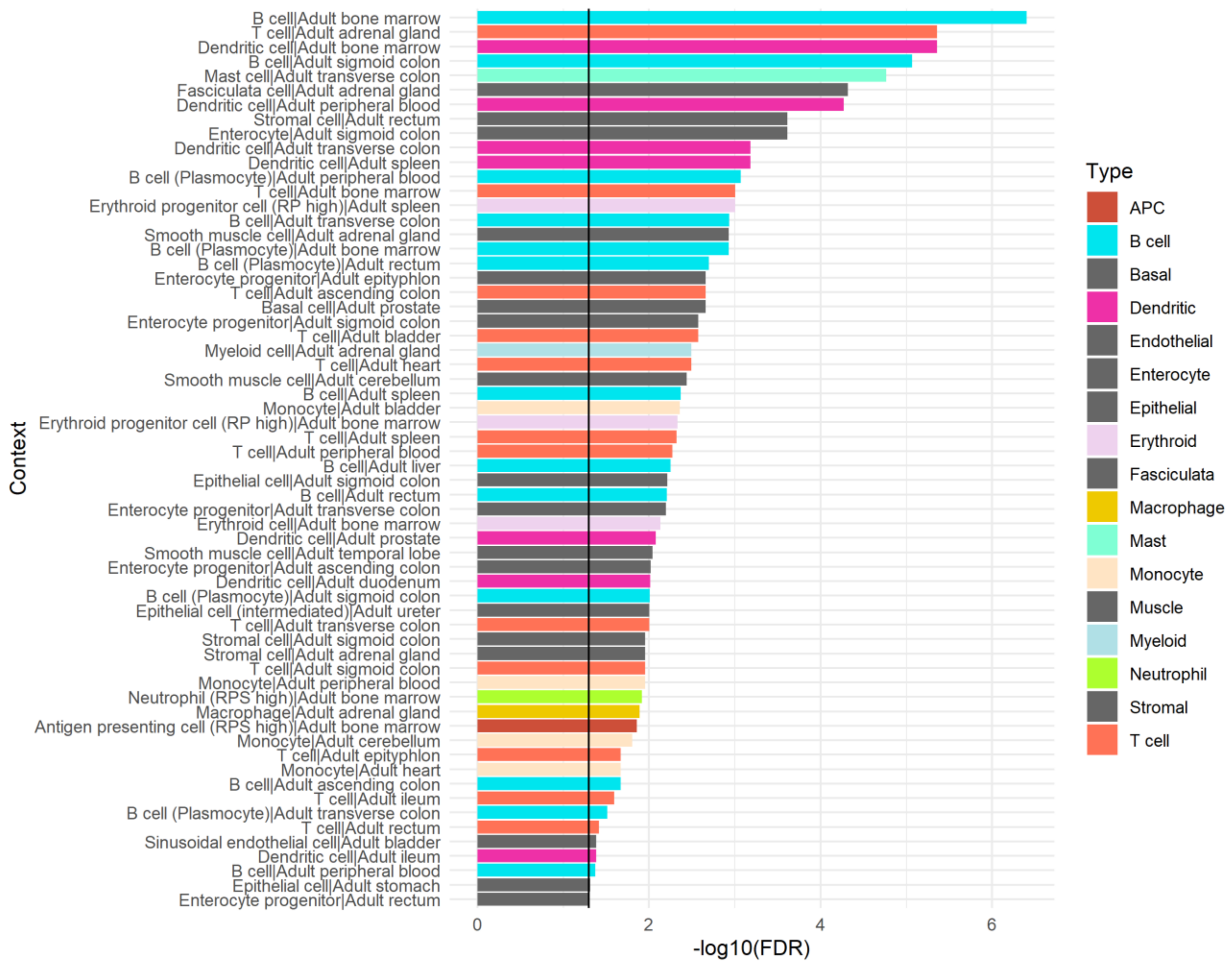

Supplementary Figure 2. Adult cell types in which membrane trafficking targets of miR-17~92 cluster are significantly less expressed relative to non-targeted genes (Supplementary Note 1, Supplementary Tables 1, 3). The vertical line signifies the 0.05 FDR threshold (one-tailed MWU test). Colored bars belong to cell types related to immune response (APC, Antigen Presenting Cell).

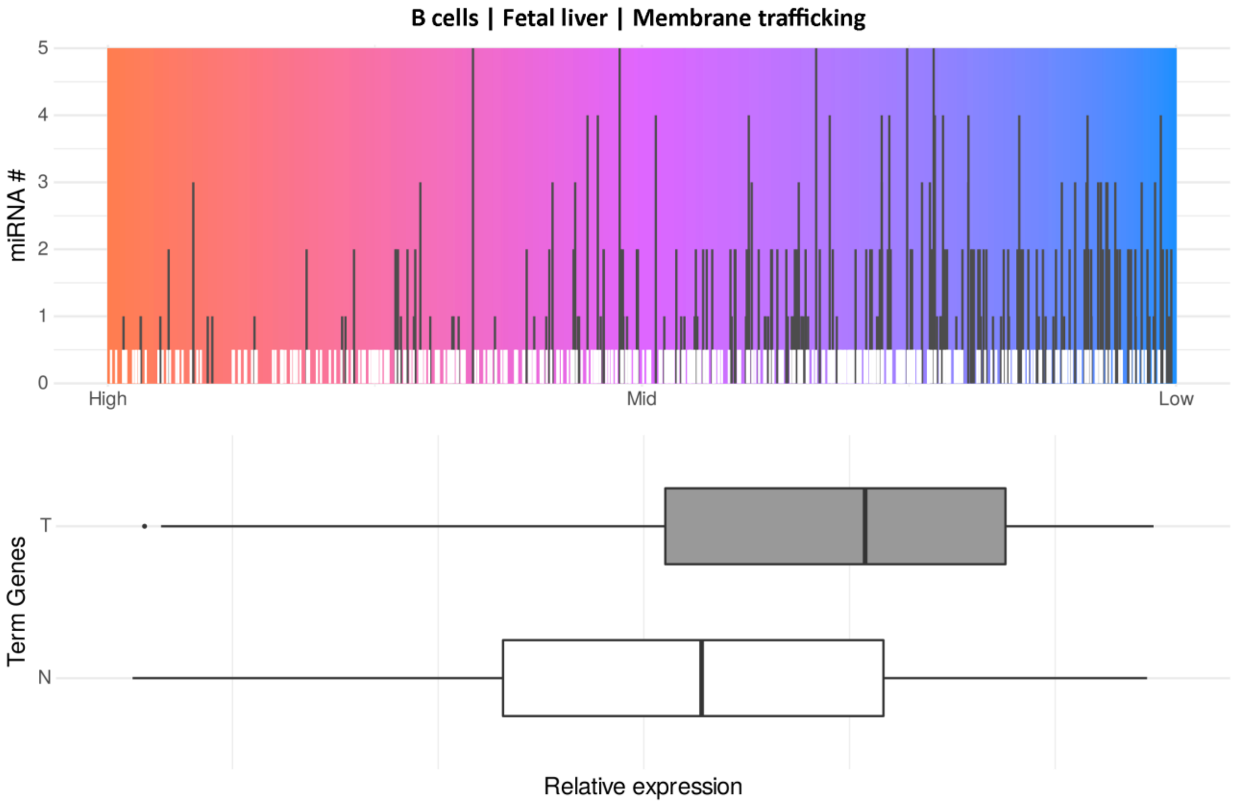

Supplementary Figure 3. Post-enrichment plot produced by miRPath for the term Membrane trafficking utilizing gene expression values from fetal liver B cells.

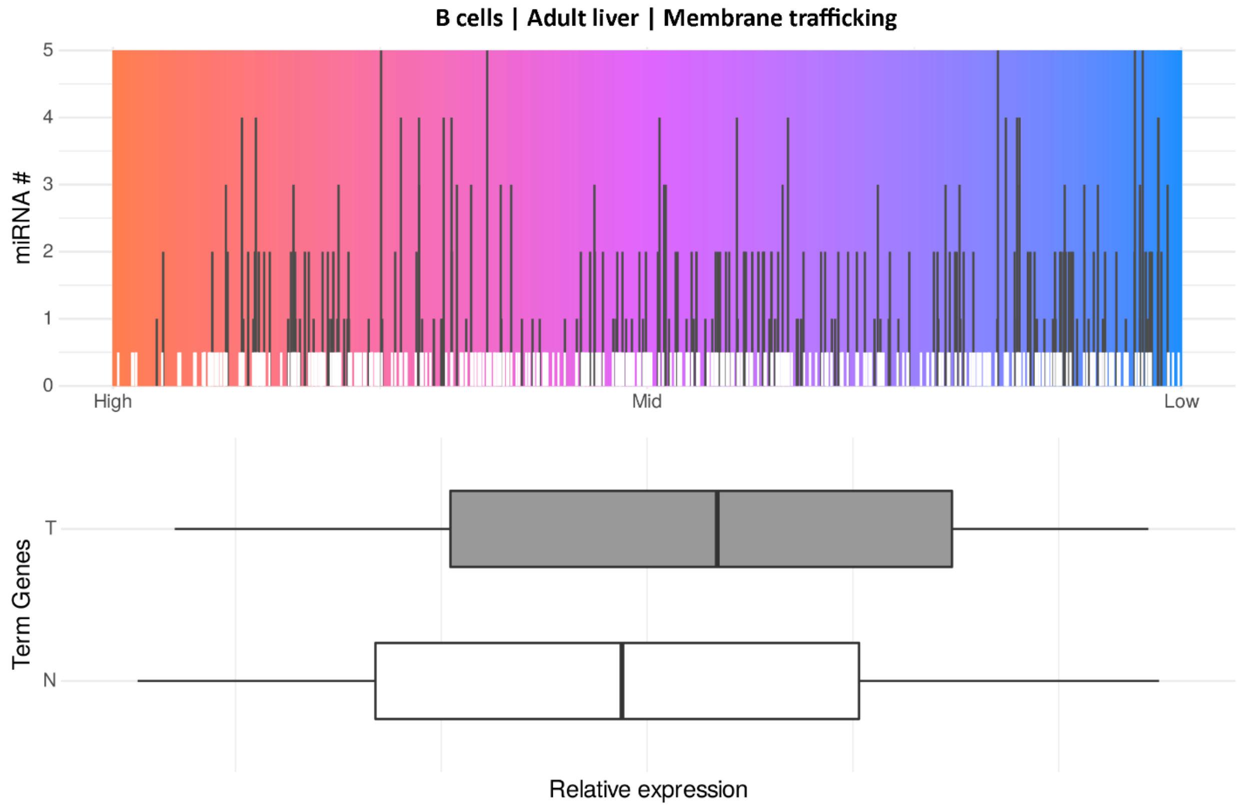

Supplementary Figure 4. Post-enrichment plot produced by miRPath for the term Membrane trafficking utilizing gene expression values from adult liver B cells.

Supplementary References

1. Mogilyansky, E. and Rigoutsos, I. (2013) The miR-17/92 cluster: a comprehensive update on its genomics, genetics, functions and increasingly important and numerous roles in health and disease. *Cell Death & Differentiation*, **20**, 1603-1614.
2. Han, X., Zhou, Z., Fei, L., Sun, H., Wang, R., Chen, Y., Chen, H., Wang, J., Tang, H., Ge, W. *et al.* (2020) Construction of a human cell landscape at single-cell level. *Nature*, **581**, 303-309.
3. Taguchi, T. and Mukai, K. (2019) Innate immunity signalling and membrane trafficking. *Current opinion in cell biology*, **59**, 1-7.
4. Yuseff, M.-I., Pierobon, P., Reversat, A. and Lennon-Duménil, A.-M. (2013) How B cells capture, process and present antigens: a crucial role for cell polarity. *Nature Reviews Immunology*, **13**, 475-486.
5. Benvenuti, F. (2016) The dendritic cell synapse: a life dedicated to T cell activation. *Frontiers in immunology*, **7**, 70.
6. Xiao, C., Srinivasan, L., Calado, D.P., Patterson, H.C., Zhang, B., Wang, J., Henderson, J.M., Kutok, J.L. and Rajewsky, K. (2008) Lymphoproliferative disease and autoimmunity in mice with increased miR-17-92 expression in lymphocytes. *Nature immunology*, **9**, 405-414.
7. Tung, Y.-T., Lu, Y.-L., Peng, K.-C., Yen, Y.-P., Chang, M., Li, J., Jung, H., Thams, S., Huang, Y.-P. and Hung, J.-H. (2015) Mir-17∼ 92 governs motor neuron subtype survival by mediating nuclear PTEN. *Cell reports*, **11**, 1305-1318.
8. Foshay, K.M. and Gallicano, G.I. (2009) miR-17 family miRNAs are expressed during early mammalian development and regulate stem cell differentiation. *Developmental biology*, **326**, 431-443.
9. Kavakiotis, I., Alexiou, A., Tastsoglou, S., Vlachos, I.S. and Hatzigeorgiou, A.G. (2022) DIANA-miTED: a microRNA tissue expression database. *Nucleic acids research*, **50**, D1055-D1061.
10. Wang, Y., Wu, J., Chen, H., Yang, Y., Xiao, C., Yi, X., Shi, C., Zhong, K., He, H. and Li, Y. (2021) Genome-wide CRISPR-Cas9 screen identified KLF11 as a druggable suppressor for sarcoma cancer stem cells. *Science advances*, **7**, eabe3445.
11. Li, W., Xu, H., Xiao, T., Cong, L., Love, M.I., Zhang, F., Irizarry, R.A., Liu, J.S., Brown, M. and Liu, X.S. (2014) MAGeCK enables robust identification of essential genes from genome-scale CRISPR/Cas9 knockout screens. *Genome biology*, **15**, 1-12.
12. Farina, A.R., Cappabianca, L., Ruggeri, P., Gneo, L., Pellegrini, C., Fargnoli, M.-C. and Mackay, A.R. (2018) The oncogenic neurotrophin receptor tropomyosin-related kinase variant, TrkAIII. *Journal of Experimental & Clinical Cancer Research*, **37**, 1-17.
13. Hechtman, J.F. (2021) NTRK insights: best practices for pathologists. *Modern Pathology*, 1-8.
14. Wilk, S.S. and Zabielska-Koczywąs, K.A. (2021) Molecular Mechanisms of Canine Osteosarcoma Metastasis. *International Journal of Molecular Sciences*, **22**, 3639.
15. Zhang, J., Yu, X.-H., Yan, Y.-G., Wang, C. and Wang, W.-J. (2015) PI3K/Akt signaling in osteosarcoma. *Clinica Chimica Acta*, **444**, 182-192.
16. Park, J.H., Shin, J.E. and Park, H.W. (2018) The role of hippo pathway in cancer stem cell biology. *Molecules and cells*, **41**, 83.
17. Wagner, V., Kern, F., Hahn, O., Schaum, N., Ludwig, N., Fehlmann, T., Engel, A., Henn, D., Rishik, S. and Isakova, A. (2023) Characterizing expression changes in noncoding RNAs during aging and heterochronic parabiosis across mouse tissues. *Nature Biotechnology*, 1-10.
